# A comprehensive mechanism for 5-carboxylcytosine-induced transcriptional pausing revealed by Markov state models

**DOI:** 10.1016/j.jbc.2021.100735

**Published:** 2021-05-13

**Authors:** Kirill A. Konovalov, Wei Wang, Guo Wang, Eshani C. Goonetilleke, Xin Gao, Dong Wang, Xuhui Huang

**Affiliations:** 1 Department of Chemistry, State Key Laboratory of Molecular Neuroscience, The Hong Kong University of Science and Technology, Kowloon, Hong Kong; 2 Hong Kong Center for Neurodegenerative Diseases, Hong Kong Science Park, Hong Kong; 3 Computational Bioscience Research Center, Computer, Electrical and Mathematical Sciences and Engineering Division, King Abdullah University of Science and Technology (KAUST), Thuwal, Saudi Arabia; 4 Department of Cellular and Molecular Medicine, University of California San Diego, La Jolla, CA, USA; 5 Division of Pharmaceutical Sciences, Skaggs School of Pharmacy and Pharmaceutical Sciences, University of California San Diego, La Jolla, California, USA

**Keywords:** 5caC, 5-carboxycytosine, FL3, fork loop 3, GMRQ, generalized matrix Rayleigh quotient, MCMC, Markov chain Monte Carlo, MSM, Markov state model, Pol II, RNA polymerase II, tICA, time-lagged independent component analysis

## Abstract

RNA polymerase II (Pol II) surveils the genome, pausing as it encounters DNA lesions and base modifications and initiating signals for DNA repair among other important regulatory events. Recent work suggests that Pol II pauses at 5-carboxycytosine (5caC), an epigenetic modification of cytosine, because of a specific hydrogen bond between the carboxyl group of 5caC and a specific residue in fork loop 3 of Pol II. This hydrogen bond compromises productive NTP binding and slows down elongation. Apart from this specific interaction, the carboxyl group of 5caC can potentially interact with numerous charged residues in the cleft of Pol II. However, it is not clear how other interactions between Pol II and 5caC contribute to pausing. In this study, we use Markov state models (a type of kinetic network models) built from extensive molecular dynamics simulations to comprehensively study the impact of 5caC on Pol II translocation. We describe two translocation intermediates with specific interactions that prevent the template base from loading into the Pol II active site. In addition to the previously observed state with 5caC constrained by fork loop 3, we discovered a new intermediate state with a hydrogen bond between 5caC and fork loop 2. Surprisingly, we find that 5caC may curb translocation by suppressing kinking of the helix bordering the active site (the bridge helix) because its high flexibility is critical to translocation. Our work provides new insights into how epigenetic modifications of genomic DNA can modulate Pol II translocation, inducing pauses in transcription.

Epigenetic modifications of the genomic DNA are part of a vast suite of regulatory machinery in higher eukaryotes. Methylated cytosine bases are epigenetic markers that cluster at CpG islands in promoters of genes, repressing their expression. Methylation is heavily regulated during development, and loss of methylation at gene promoters is linked with cancer (1, 2). The 10–11 translocation methylcytosine dioxygenases mediate the removal of the methyl group from cytosines in three consecutive oxidation reactions. The intermediate species of this pathway are stable and can be detected, for instance, in mammalian nuclear extracts (3). The last intermediate, 5-carboxycytosine (5caC), transiently pauses a transcribing RNA polymerase II (Pol II) (4). Promotor-proximal pausing of Pol II is critical to a variety of cellular processes, including gene regulation and DNA repair (5). Although pausing of RNA polymerases has been studied and extensively reviewed (6, 7, 8), the detailed mechanism of Pol II pausing with 5caC remains to be revealed.

Based on biochemical assays and crystal structures of the yeast Pol II elongation complex, Wang *et al.* suggested that 5caC reduces transcription by forming a hydrogen bond between its carboxylic modification and the side chain of a glutamine residue in Pol II (Q531 of the subunit Rpb2) (9). Residue Q531 is located on fork loop 3 (FL3), which is also termed the epi-DNA-recognition loop. Structures of the Pol II elongation complex in the post-translocation state, with an unmodified cytosine (C) paired with GTP (PDB ID: 2E2H), show that the template base is located at the +1 site, and the side chain of Q531 does not directly interact with the base (10). In contrast, in a structure of Pol II elongation complex with 5caC (PDB ID: 4Y52), the electron density of the 5caC suggests that two states are present: a canonical post-translocation state and an intermediate state in which the base is held by a hydrogen bond with Q531 in a midway position between the +1 and +2 template sites. This midway state is believed to reduce the catalytic efficiency of Pol II because in another structure with 5caC and a GTP analogue bound in the active site (PDB ID: 4Y7N), the midway 5caC base pulled the GTP analogue away from the canonical bound pose. The biochemical data show a 4.2-fold reduction in GTP incorporation specificity (4), whereas the Q531A mutation leads to a 2.6-fold increase (9). Because this mutation only partially rescues transcription with 5caC, an additional mechanism may contribute to 5caC-induced pausing. Apart from the interaction with Q531, the carboxylic group of 5caC can potentially interact with numerous charged residues in the cleft of Pol II and induce metastable translocation intermediates that would compromise correct template loading of 5caC into the canonical +1 site. In this work, we sought to survey the translocation dynamics of 5caC during template loading and analyze the transient translocation intermediates induced by 5caC and their impact on Pol II translocation.

Previous studies explored the intermediates of both forward and backward translocation (11, 12) and have highlighted that bridge helix fluctuation is a major driving force for the transition between these intermediates. The bridge helix is a universally conserved (Fig. S1*A*) metastable α-helix that separates the upstream DNA:RNA duplex in the active site from the downstream DNA duplex. During translocation, the template nucleotide base crosses over the bridge helix to reach the active site (+1 template position); this process is accompanied by vigorous thermal fluctuations of the central part of the bridge helix, as seen in molecular dynamics (MD) simulations (11, 12, 13). The bridge helix can deviate from an idealized straight α-helix by partially uncoiling and kinking, meaning that the helix has distinct bending points connected by stretches of relatively straight helical segments. Experimentally determined structures of RNA polymerases support the significance of bridge helix kinking (10, 14, 15). Several inhibitors of the RNA polymerases were shown to form contacts with the bridge helix, limiting its mobility: streptolydigin and a class of N-hydroxy-N′-phenyl-3-trifluoromethyl-benzamidine RNAP inhibitors in the bacterial RNA polymerase and α-amanitin in Pol II (16, 17, 18, 19). A kinked bridge helix was resolved in a structure of paused bacterial RNA polymerase (14), where the bridge helix protruded into the active site and partially obstructed the NTP-binding site. Moreover, the work of Tan *et al.* (20) describes archaeal RNA polymerases with systematically mutated residues of the bridge helix and shows that depending on the mutation, RNA polymerases can transcribe either slower or faster than the WT. Flexibility of the N terminus of the bridge helix was suggested to play an important role in transcription (21). A recent structural study demonstrated that the latch domain of the elongation factor RTF1 connects with the N terminus of the bridge helix and may be responsible for allosterically stimulating elongation by enhancing bridge helix flexibility (22). It is evident that the bridge helix can assume both the straight and kinked conformations and can be modulated to either enhance or curb translocation. Therefore, altered bridge helix mobility may lead to reduced translocation.

MD simulations can complement structural experiments by resolving the dynamics and identifying transient molecular conformations at atomic resolution. However, slow conformational changes of larger molecular assemblies, such as Pol II, require special techniques to correctly estimate the kinetic and thermodynamic properties of the system under limited sampling. Markov state models (MSMs) are statistical models that can be used to estimate conformational changes observed in MD simulations as Markovian events take place at discrete timesteps (23, 24, 25, 26, 27, 28, 29, 30, 31, 32, 33) and can overcome the limitations of MD simulations by bridging the timescale gap inherent to conventional MD simulations. MSMs can also be estimated from multiple independent MD simulations, allowing the sampling to be conducted in parallel, a highly desirable trait with modern supercomputers and distributed computing systems (34, 35) as it greatly expands the range of accessible timescales. MSMs have been widely used to study the conformational changes of biomolecules (11, 12, 35, 36, 37, 38, 39, 40, 41, 42, 43, 44, 45, 46, 47, 48). For Pol II, both forward translocation and backward translocation upon misincorporation (backtracking) were studied with MSMs estimated from MD simulations. Silva *et al.* studied the forward translocation of yeast Pol II and highlighted the pivotal role of residue Y836 located on the C terminal of the bridge helix (11). Da *et al.* (12) modeled the backward translocation of Pol II and found that the oscillation of the middle section of the bridge helix plays a critical role in this process. These studies establish that both forward and backward translocation proceed through a series of previously unobserved metastable intermediates. Thus, the MSMs derived from MD simulations have the potential to reveal the details of Pol II translocation with 5caC that may have eluded previous experiments.

In this work, we studied Pol II translocation using MSMs based on MD simulations to resolve the intermediate states that pause transcription with 5caC. Because 5caC differs from C by a single carboxyl group located on the base of the nucleotide, we expect the major differences to arise during base translocation. Hence, we focused our simulations on the post-translocation free energy basin: with the nucleic acid backbone in the post-translocation state and the base of the template nucleotide transitioning between the +1 and +2 template sites (Fig. 1*A*). We observed that the carboxylic group of 5caC forms numerous hydrogen bonds with Pol II and the most stable interaction is formed with the Q531 residue of the epi-DNA-recognition loop, consistent with previous results (9). In addition, we found a novel interaction capable of reducing transcription: R512 of Rpb2 can form a stable ion pair with the carboxylic modification, which restricts 5caC from moving into the active site. This residue is conserved across all domains of life (Fig. S1*B*) and is likely involved in multiple steps of transcription: initiation, translocation, and catalysis (49, 50, 51). Interestingly, the bridge helix kinks less with 5caC, likely slowing the transition of the template nucleotide into the active site. The kinking pattern is altered throughout the length of the bridge helix, even at regions distal from the middle section, where the 5caC base contacts it, indicating that 5caC indirectly alters kinking. We conducted additional MD simulations with mutant variants of Pol II that lack specific interactions with 5caC and found that each of these mutations promotes translocation in distinct ways: Q531A reduces the energy barrier for the base transition between the midway and the +1 template site, whereas R512A favors forward translocation of the base from the +2 site into the midway state. In addition, we found that the R512A mutation restores bridge helix kinking. Therefore, we concluded that 5caC exhorts a double effect on the Pol II: in addition to pausing in the midway state constrained by hydrogen bonds with residues of Pol II and misaligning the NTP bound in the active site, it indirectly reduces bridge helix kinking, slowing down the transport of the template base into the active site.

**Figure 1:**
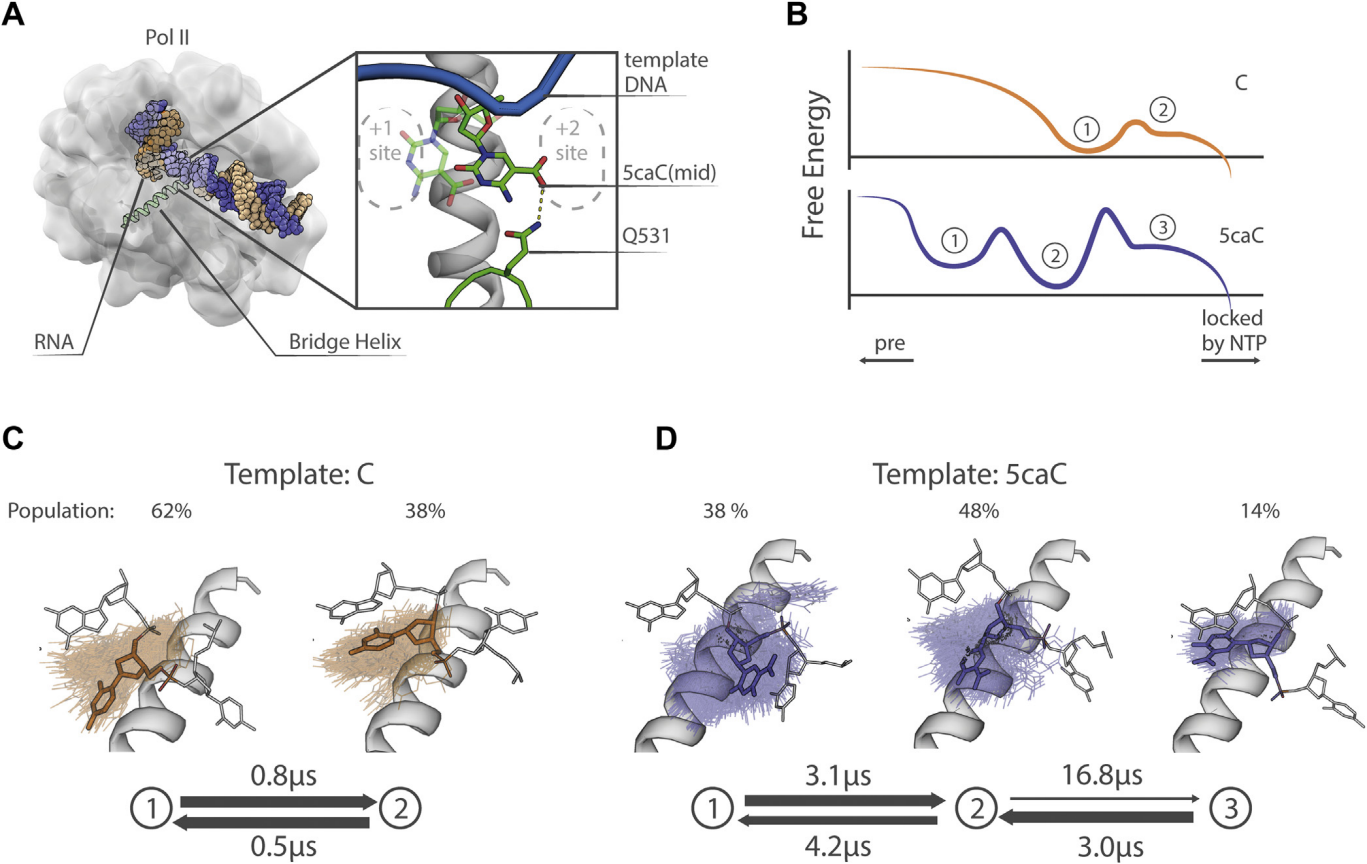
**Overview of the Pol II elongation complex with 5caC as the template nucleotide in the midway position.***A*, overview of the simulated Pol II elongation complex. The *box* highlights the two conformations of 5caC previously detected in the crystal structure with PDB ID: 4Y52. *B*, schematic free-energy plot of the translocation intermediates identified with MSMs. *C*, the transition pathway of the elongation complex with C as a template. *D*, the major transition pathways (at least 95% of overall flux) of the elongation complex with 5caC as a template. Conformations of the template nucleotide base sampled from each macrostate are shown with a *transparent color* over the ideal bridge helix shown in *white*. A representative orientation of the template nucleotide is shown with *colored sticks* together with the adjacent upstream and downstream nucleotides shown in *white*. The equilibrium populations are shown above each state. 5caC, 5-carboxycytosine; MSMs, Markov state models; Pol II, RNA polymerase II. (See Tables S1 and S2 for more precise mean and error estimates).

## Results and discussion

### Markov state models reveal additional intermediate states of Pol II translocation induced by 5caC

To investigate how 5caC pauses the elongation complex, we conducted MD simulations starting with a combined structure based on a model from our past study (11) and a previously reported crystal structure with PDB ID: 4Y52, which captured two orientations of 5caC: midway and insert (9). Both orientations were used to initiate the simulations (Fig. 1*A*). To distinguish the effect of 5caC from C, we also initiated MD simulations with the same two structures and changing 5caC to C. With each of these four starting structures, we performed two rounds of MD simulations to sample the conformations in the post-translocation basin (10-μs simulation time for C and 5caC combined) and calculated MSMs for C and 5caC utilizing our previously established protocol (52). To faithfully reproduce the kinetics, several parameters of the MSM require optimization: the feature set used for dimensionality reduction, number of microstates, and so forth. We selected a feature set based on interatomic distances to describe translocation (see Experimental procedures for details), totaling 1481 atom pairs from 5caC, neighboring residues of the bridge helix, FL3, and template DNA nucleotides. The structural elements of Pol II and the selected pairs of atoms are illustrated in Figure S2. With this feature set as input, we performed a time-lagged independent component analysis (tICA) (53, 54, 55) to reduce the dimensionality to the first two components (tIC1 and tIC2) and selected the tICA lag time with a cross-validation scheme based on the generalized matrix Rayleigh quotient (GMRQ) (56) (Fig. S3*A*). We then grouped all MD conformations into 800 microstates (a number that yielded the highest GMRQ score out of the tested choices, Fig. S3*B*) using the K-centers algorithm (57) and separately projected the individual datasets (5caC and C) onto the clusters to calculate two MSMs (lag time of 45 ns, chosen based on leveling of the timescales Fig. S3, *C* and *D*). We further validated both models using a residence probability test (Fig. S4). Refer to the Experimental procedures section for further details on the simulation setup and MSM construction.

Our MD simulations sampled the conformational space close to the post-translocation state (Fig. 1, *A* and *B*), where the template nucleotide base can point to either side of the bridge helix without significant backbone translocation of the template DNA. Our MSM for C identifies two metastable states (Fig. 1*C*), while our MSM for 5caC identifies three metastable states (Fig. 1*D*). For C, the template base in both metastable states occupies the +1 template site, pointing toward the active site (Fig. 1*C*). State 1 is distributed diffusely compared with state 2, where the template nucleotide base is tightly stacked with the upstream DNA base. Because the base points into the active site in both of these metastable states, they are expected to support canonical pairing with an NTP and subsequent catalysis. Examining the states of 5caC, we observed that the base of the template nucleotide in state 1 lies closer to the +2 template site (the right of the bridge helix in Fig. 1*D*) and displays a broad distribution of orientations. In state 2, the conformations of 5caC spread out over the bridge helix, indicating a different–midway–state. State 3 of 5caC resembles state 2 of C, with the base pointing toward the active site and tightly stacking with the upstream base. State 2 of 5caC is energetically the most favorable, representing close to 50% of the total population, while the populations of states 1 and 3 are estimated to represent 38% and 14% of the total population, respectively. The significant populations of states 1 and 2 of 5caC, in which the base of the template nucleotide is shifted away from the active site, may pause transcription by misaligning the incoming NTP or by preventing pairing altogether.

To learn more about the interconversion of these states, we estimated the kinetic network of the sampled structures using the transition path theory and calculated the mean first passage times between states (Table S2). The two metastable states of C take less than a microsecond to interconvert (Fig. 1*C*), whereas the macrostates of 5caC, which are arranged along a single unbranching path, transition between states at an order of magnitude slower than C. Transitions out of state 2 are the slowest, indicating a kinetic bottleneck in the translocation (Fig. 1*D*). The transition from state 2 into state 3 is an order of magnitude slower (close to 17 μs) than C. Hence, during translocation, 5caC necessarily passes through an energetically favorable state 2, with its base oriented along the axis of the bridge helix, and transitions out of this state are slow compared with normal translocation.

For C, the interconversion of metastable states 1 and 2 is on the order of microseconds, which agrees with a previous MSM study of translocation (11). The study also shows that the movement of the template DNA backbone is the slowest step during translocation, taking up to tens of microseconds, whereas the base transition proceeds at a faster rate, on the order of microseconds. Our estimate of the 5caC base translocation rate is slower: it reaches an order of tens of microseconds for the rate-limiting transition, which is consistent with experimentally observed reduced transcription (4).

### 5caC reduces bridge helix kinking to slow down the translocation

**Figure 2:**
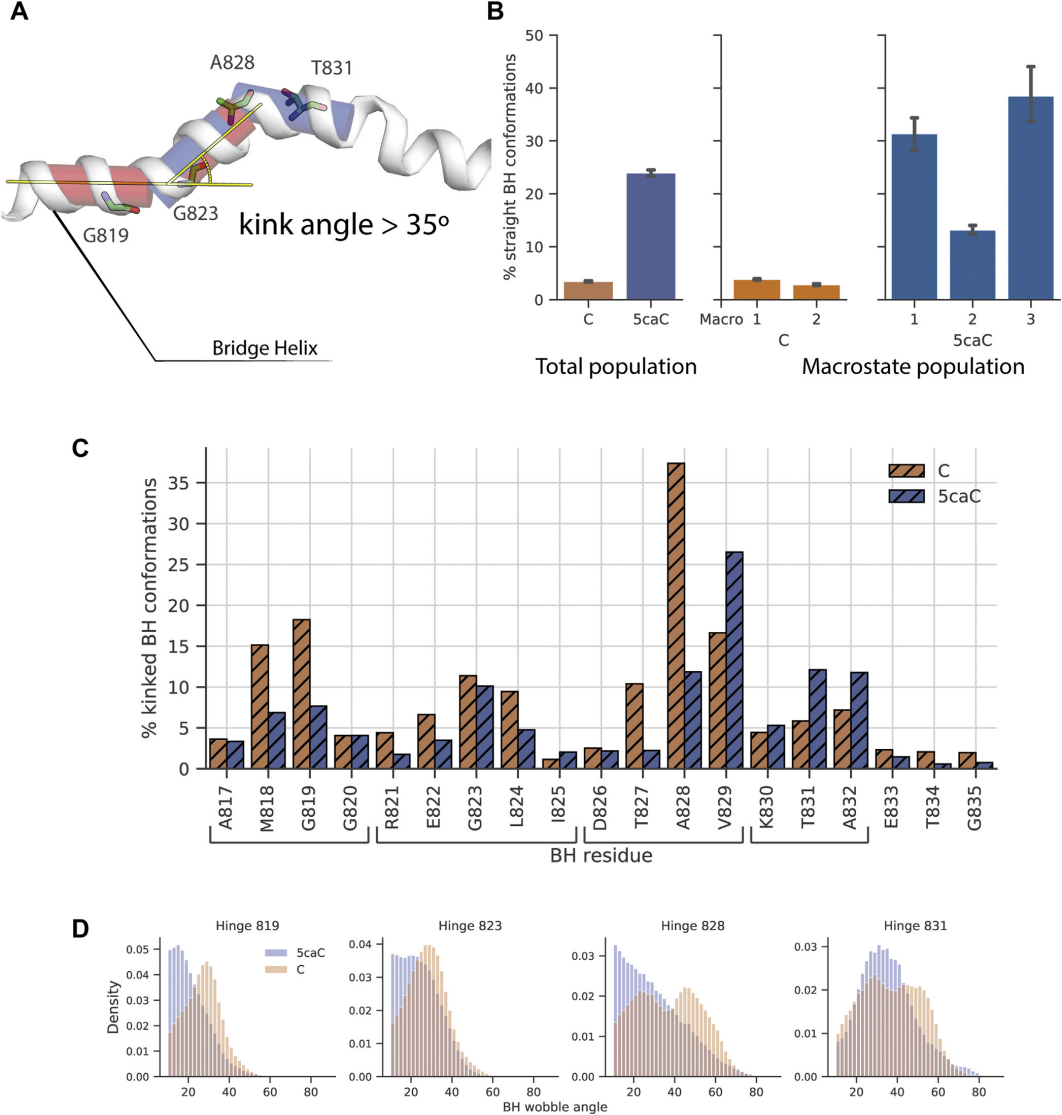
**Bridge helix bending is reduced with 5caC, compared with C.***A*, overview of the Kink Finder algorithm applied to the bridge helix: cylinders fit in consecutive residues are shown in a *transparent color*, and the measured kink angle is illustrated by *yellow lines*. Hinge center residues are labeled. *B*, the number of straight conformations (kink angle <35*°*) in 5caC and C in the overall population (*left*) and in individual macrostates of C (*middle*) and 5caC (*right*). The mean and error (95% of the bootstrap distribution, over 1000 resamples) estimates are calculated from ten Markov chain Monte Carlo (MCMC) trajectories of 10^5^ steps generated from the MSM. *C*, bridge helix kinking calculated for individual residues. Compared with *panel B*, this panel uses hatching to depict kinked bridge helix conformations, rather than straight. *D*, normalized histogram of wobble angles aggregated over the hinges. The hinges include the following residues: hinge 819 includes residues 816 to 820, hinge 823: 821 to 825, hinge 828: 826 to 828, hinge 831: 829 to 832. 5caC, 5-carboxycytosine; MSM, Markov state model.

In this section, we describe how the kinking of the bridge helix is altered with 5caC. The distribution of bridge helix kink angles (as measured by Kink Finder (58) illustrated in Fig. 2*A*, see Experimental procedures for details) revealed substantial differences between C and 5caC, especially at residues 825 to 828 (Fig. S5). The majority of the bridge helix conformations are kinked in both the C and 5caC systems, but 5caC displays significantly more straight conformations—close to 25% of the overall population (Fig. 2*B*, left). The hydrogen bonds between the backbone between the i and i + 4 residues support the helical structure and are commonly missing in kinked structures (58). Straight conformations are rigidified by the hydrogen bonds between the backbone atoms preventing significant fluctuations; thus, reduced kinking of the bridge helix implies reduced translocation. We examined how the bridge helix kinks in individual macrostates and reported the percentage of straight bridge helix conformations in the total population in each macrostate (Fig. 2*B*, the middle and right). The elongation complex with C displays an approximately equal percentage (close to 5%) of straight conformations in each of its states. Conversely, kinking varies across the metastable states of 5caC: the bridge helix is straight in roughly 30% of the population in states 1 and 3, whereas in state 2, the percentage of straight conformations is half that amount (close to 15% of the population). States 1 and 2 account for roughly 75% of straight conformations in the overall population of 5caC. With state 3 being the least populated (14% of the total population, Fig. 1*D*), and the base already in the +1 site, kinking of the bridge helix in this state likely does not impact transcription. This observation shows that states 1 and 2 of 5caC contribute to Pol II pausing by restricting bridge helix kinking.

The distribution of the kinks over the length of the bridge helix is similar between C and 5caC (Fig. 2*C*). Although there appears to be a large difference between 5caC and C at residues A828 and V829, we dismiss it because the kinks at neighboring residues lead to similar structures and we later group residues into hinges. We note that kinks cluster at four regions centered at the following residues: G819, G823, A828, and T831. Kinks are also frequently observed in crystal structures of Pol II, often several at once: PDB ID 2E2H (10) has kinks at residues 826 and 832; PDB ID 3I4N (59)–819, 825, 834; PDB ID: 3PO3 (60)–at residues 819, 829; PDB ID 3CQZ (17): 819 and 832 (see Fig. S6 for the kinking profiles measured with Kink Finder). Apart from kinks seen in the crystal structures, our simulations reveal significant kinking around residue G823. We grouped kinks measured at individual residues into hinges (see the caption to Fig. 2) based on the similarity of wobble angle distributions of individual residues (Fig. S5). Compared with C, 5caC shifts the peak of kink angle distributions for hinges 819 and 823 to lower magnitudes, indicating limited flexibility of this segment of the bridge helix (Fig. 2*D*). The residues around hinge 819 are reported to be critical for transcription as their mutations produce both defective and hyper-transcribing polymerases (20). Also, α-amanitin bound to Pol II contacts residues surrounding A817, likely restricting bridge helix flexibility at hinge 819 (17). Thus, 5caC can reduce transcription by restricting kinking at the N terminus of the bridge helix. With C, the hinges centered at residues 828 and 831 have two stable conformations, which are evident from two peaks close to 25° and 50° in the kink angle distribution. With 5caC, hinge 831 has a single stable kinked conformation (indicated by the peak at 30°), whereas the distribution of kink angles at hinge 828 is decaying monotonically, meaning that both kinked conformations at this hinge become unstable. When crossing over the bridge helix, 5caC primarily contacts the central region of the bridge helix (residues 828–835), so the kinking of hinges at residues 819 and 823 is reduced indirectly, through the bridge helix itself or the neighboring Pol II residues.

Altogether, the bridge helix kinks less frequently with 5caC compared with C. Kinks cluster around four positions (hinges) in the bridge helix, and 5caC either reduces the magnitude of a kink or abolishes a stable kinked conformation, depending on the hinge. The differences between C and 5caC provide a possible explanation for transcription slowdown: 5caC restricts bridge helix kinking throughout its length, stabilizing it in a straight conformation, preventing efficient translocation of the template nucleotide into the active site.

### 5caC forms specific hydrogen bonds in the midway states

**Figure 3:**
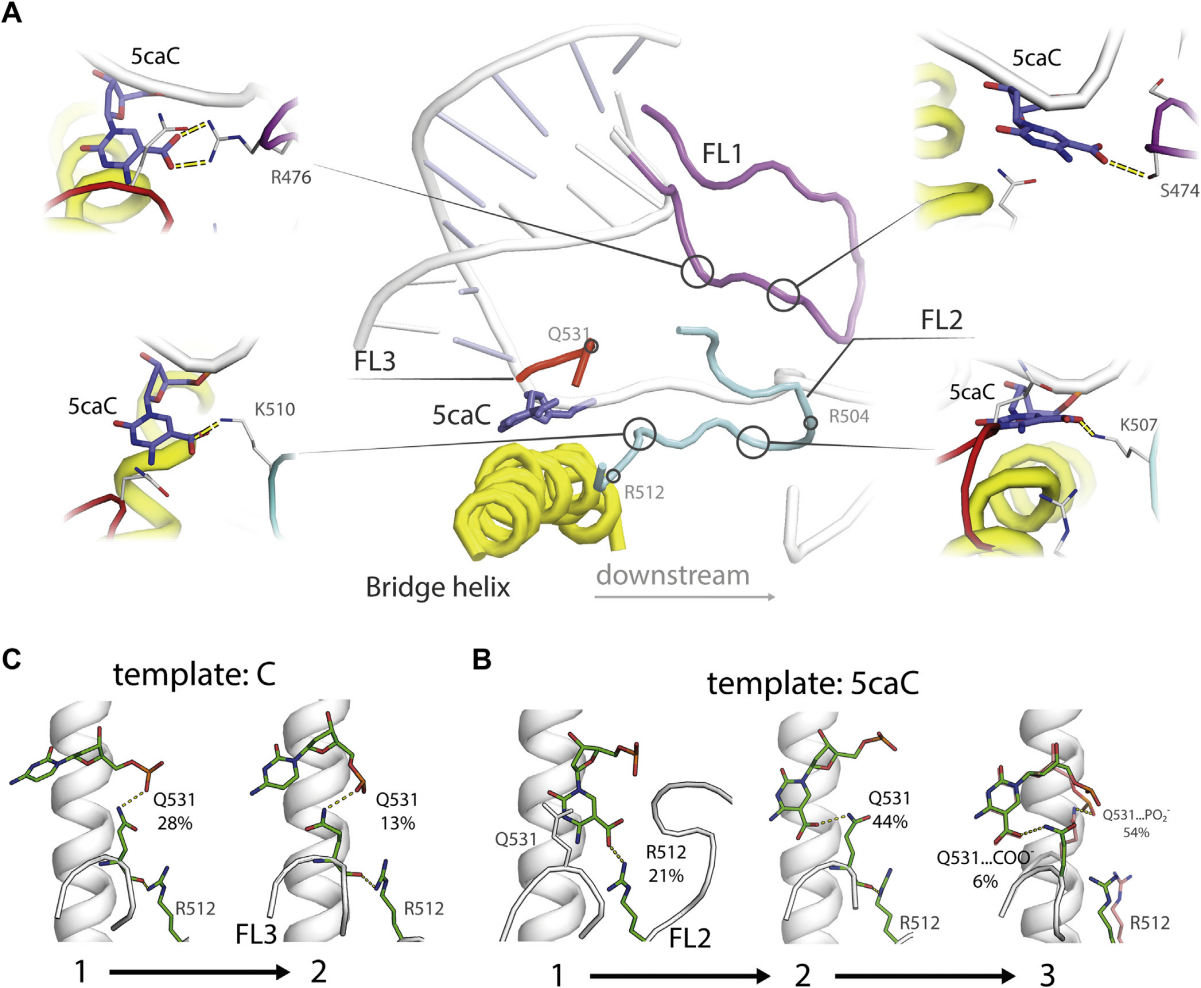
**Major interactions between the template nucleotide and Pol II.** Major interactions between the template nucleotide and Pol II. *A*, overview of Pol II structural elements (*middle*). Locations of residues that form hydrogen bonds with 5caC are highlighted with *circles*. *B*, dominant interactions of the 5caC modification with residues of Pol II in each of the macrostates. An alternative conformation is shown with a dominant but non-5caC-specific hydrogen bond between Q531 and the phosphate moiety of 5caC. The mean population of the hydrogen bond in the macrostate is shown. *C*, interactions of C with the protein residues in macrostates corresponding to the interactions of 5caC. 5caC, 5-carboxycytosine; Pol II, RNA polymerase II.

The carboxylic group of 5caC forms stable hydrogen bonds (occurring in >5% of the macrostate population) with residues in fork loops 1 to 3 of Pol II: FL1 (Rpb2^470-480^), FL2 (Rpb2^502-509^), and FL3(Rpb2^521–541^) (Fig. 3*A*). We reported the percentage of conformations in each metastable state that exhibit a given hydrogen bond. Overall, 5caC forms more hydrogen bonds than C, both in terms of the percentage and the number of possible bonding partners (Tables S3 and S4). For 5caC, states 1 and 2 display the most hydrogen bonds, especially with the carboxylic modification. The percentage of conformations with hydrogen bonds between residues of FL1, 2, and 3 and the carboxylic modification of 5caC add up to 57% of the population in state 1 and 84% of the population in state 2 (Fig. S7). These residues are located on the downstream side of the bridge helix (except for Q531, which is located directly above the bridge helix); therefore, all these bonds can prevent 5caC from reaching the active site. The large population of hydrogen bonds in state 2 of 5caC can explain the slow transition into state 3. Upon reaching state 3, however, the hydrogen bonding with the carboxylic modification reduces to the level where it is no longer dominating among other hydrogen bonds (25% of the macrostate population, Fig. S7); therefore, from both structural perspective and hydrogen bonding, state 3 of 5caC does not differ from the post-translocated state of C.

The side chain of R512 (Rpb2) forms the most prominent hydrogen bond with the carboxylic modification in state 1 (Fig. 3*B*), occurring in 21% of the macrostate. However, in states 2 and 3, the hydrogen bond between R512 and 5caC is insignificant. In state 2, the most stable hydrogen bond with 5caC forms between its carboxylic modification and the side chain of Q531 (44% of the state population). This bond was previously detected in the crystal structure of Pol II that was used to construct the initial structure for our simulations (PDB ID: 4Y52) (9). This hydrogen bond forms much less frequently in state 1 (8%), likely because of an unfavorable orientation of the Q531 side chain and some conformations having the FL3 reside on top of 5caC (as seen in Fig. 3*B*, state 1). In state 3, the Q531 residue predominantly interacts with the phosphate moiety of 5caC, being present in 54% of the state population, while bonding with the carboxyl group accounts for just 6% of the state population. To quantify the effect of hydrogen bonding on bridge helix kinking, we calculated the odds ratio of hydrogen bonds to occur with a straight bridge helix *versus* a kinked bridge helix. The hydrogen bonds between 5caC and R512 show a higher probability to coincide with a straight bridge helix rather than a kinked bridge helix, as illustrated in Figure S8. Conversely, the Q531 hydrogen bond has a less-significant effect on bridge helix kinking, having an odds ratio closer to 1. Therefore, the hydrogen bond between 5caC and R512 supports straight conformations of the bridge helix, and Q531 likely does not affect bridge helix kinking significantly, explaining the low percentage of straight bridge helix conformations in state 2 (Fig. 2*B* right).

Examining how C differs from 5caC, we noted that C does not interact with R512; instead, the side chain of R512 predominantly interacts with the backbone of Q531 (this hydrogen bond can be seen in crystal structures, such as PDB ID: 2E2H (10)). The side chain of Q531 interacts with the phosphate of the template nucleotide in both metastable states of C; however, this interaction is significantly less stable compared with 5caC and occurs in 28% and 13% of the population of states 1 and 2, respectively (Fig. 3*C*). The dominant interaction with C in state 2 is between the backbone of the template nucleotide (phosphate and deoxyribose) and R839 located on the C terminus of the bridge helix, occurring in 33% of this state. We also detected this hydrogen bond with 5caC, occurring in 6, 30, and 41% in states 1, 2, and 3, respectively. As C proceeds from state 1 to state 2, the hydrogen bonding between R839 and the sugar moiety of the template nucleotide increases, and the hydrogen bonding with Q531 decreases. Similarly, with 5caC, the population of states with hydrogen bonding between R839 and deoxyribose increases as the complex progresses through states 1, 2, and 3 (Fig. S7). The R839 hydrogen bond in state 2 of C and state 3 of 5caC may restrict the template nucleotide fluctuations and explain the tight stacking of the template nucleotide with the upstream DNA base (as illustrated in Fig. 1, *C* and *D* states 2 and 3, respectively). This residue likely plays a role in transcription because when mutated to alanine, the homologous R829 in *Methanocaldococcus jannaschii* reduces RNAP activity (20). We speculate that through hydrogen bonding with the phosphate group of the template DNA, Q531 may play a role in translocation by balancing the attractive force from the C terminus of the bridge helix that arises between the positively charged residues (R839 and R840) and the negatively charged backbone of the template DNA.

Because hydrogen bonds specific to the carboxylic group of 5caC occur downstream and midway to the +1 template site, they render it unfavorable for the 5caC base compared with the midway position, reflected in the high population of state 2 and low population of state 3 as well as the slow transition into state 3. The disrupted balance in the non-5caC-specific interactions may further decrease the translocation rate. Moreover, the hydrogen bond between 5caC and R512 reduces bridge helix kinking and likely slows down translocation. This mechanism is supported by previous studies that explored bridge helix flexibility in both forward and backward translocation (11, 12). The hydrogen bond with Q531 was evident from the crystal structure, and Wang *et al.* (9) showed that its mutation to alanine partially restores transcription with 5caC. However, the hydrogen bond with R512 has not been previously reported and can also contribute to pausing at 5caC.

### *In silico* mutations of Rpb2 Q531 and R512 promote translocation with 5caC

To further examine the role of residues R512 and Q531 in slowing down translocation with 5caC, we mutated these residues to alanine and conducted additional MD simulations. If we choose the initial structure of the simulation close to the transition region between metastable states in the WT protein (*i.e.*, the system will transition to both of the closest states with equal probability), then the mutants that are incapable of forming the hydrogen bonds that slow down translocation should favor the base transition toward the +1 template site. Because the hydrogen bond between 5caC and R512 appears only in state 1, we initiated a new round of simulations with R512A mutation, selecting ten starting structures closest to the barrier between states 1 and 2. Similarly, because the interactions between 5caC and Q531 predominantly appear in state 2 (Fig. 3*B*), to estimate the effect of the Q531A mutation, we started the simulations close to the barrier between states 2 and 3.

**Figure 4:**
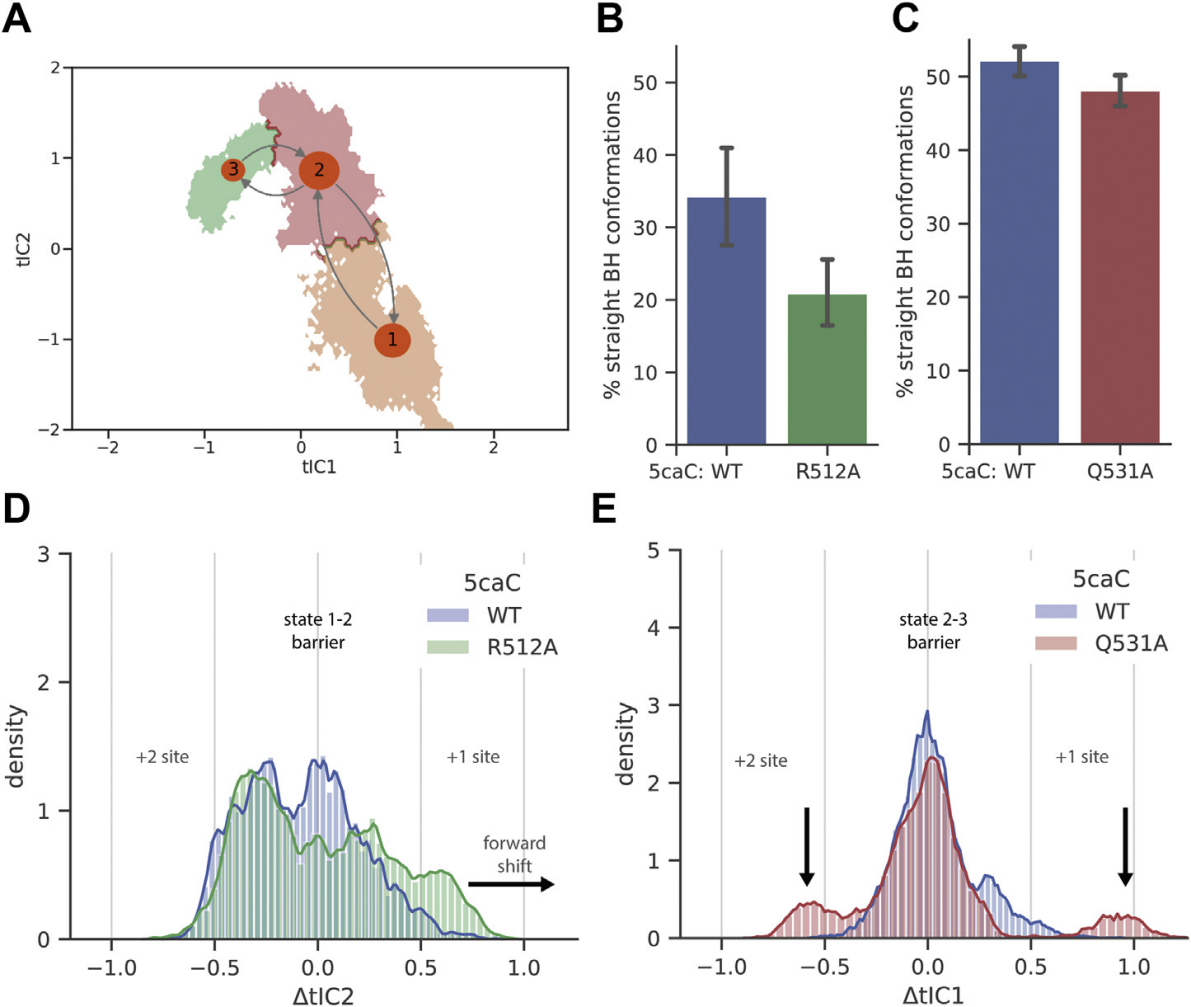
**Mutations in Pol II promote translocation and restore bridge helix bending.***A*, tICA projection of the 5caC simulation; the transition path is shown with *arrows*. The state label size reflects the population of the state. *B*, the percentage of the straight bridge helix conformation in the R512A simulation. *C*, the percentage of the straight bridge helix conformation in the Q531A simulation. The mean and error (95% percentile of the bootstrap distribution) in *panels B* and *C* were produced by bootstrapping blocks of 4 ns 1000 times with replacement. *D*, the normalized histogram of the R512A mutant simulations along the second tIC, relative to the initial conformation. The *arrows* indicate the notable differences between the mutant and the WT. *E*, the normalized histogram of the Q531A mutant simulations along the first tIC, relative to the initial conformation. 5caC, 5-carboxycytosine; Pol II, RNA polymerase II; tIC, time-lagged independent component; tICA, time-lagged independent component analysis.

To assess the progress of Pol II along the translocation coordinate, we measured the shift from initial conformations along tICA coordinates (tIC1 and tIC2) in which the MSM was built. As shown in Figure 4*A*, the projection of MD conformations onto tIC1 and tIC2 indicates that the transition from state 1 to 2 involves a large change along tIC2 and a smaller change along tIC1. Therefore, we monitored the change of tIC2 to assess the effect of the R512A mutant simulations initiated from the free-energy barrier separating states 1 and 2. As shown in Figure 4*D*, the R512A mutant simulations display a larger positive shift of tIC2 than the WT simulation, indicating that this mutant favors the transfer of the base forward along the translocation coordinate. Moreover, the bridge helix in this mutant also displays fewer straight conformations than the WT (Fig. 4*B*). These observations suggest that the R512A mutation favors forward translocation: from state 1 to state 2. Similarly, we monitored the change of tIC1 to assess the effect of the Q531A mutation, as the transition from state 2 to 3 proceeds mostly along the change of tIC1 (Fig. 4*A*). As shown in Figure 4*E*, the Q531A mutant simulation produces a smaller central peak along ΔtIC1 and two additional peaks in both directions: toward the +1 and +2 template sites. This observation suggests that the Q531A mutation destabilizes the transition state and reduces the free-energy barrier between states 2 and 3. However, unlike R512A, the Q531A mutant does not favor translocation toward the +1 or +2 template sites. The bridge helix in the Q531A mutant displays insignificantly fewer straight conformations than the WT protein (Fig. 4*C*), consistent with our analysis of bridge helix kinking and hydrogen bonds in the previous section (Fig. S8).

The results above demonstrate that R512A and Q531A mutations can improve the translocation with 5caC in two distinct ways: R512A makes the forward state (state 2) more favorable than state 1 (Fig. 4*D*), and Q531A reduces the free-energy barrier between states 2 and 3 without changing the stability of the states (Fig. 4*E*). In addition, the R512A mutation restores bridge helix kinking, potentially promoting translocation (Fig. 4, *B* and *C*).

In light of previous experimental studies, we can rank the relative transcriptional activities of the R512A mutant and WT Pol II on unmodified or 5caC-containing template. On the one hand, previous experiments showed that the R512A mutant slows down Pol II transcription by 10-fold on unmodified templates in comparison with WT Pol II, possibly because of allosteric interactions (51). On the other hand, Kellinger *et al*. (4) showed that the presence of 5caC reduces (the catalytic rate constant - kpol) almost 100-fold, compared with an unmodified template for WT Pol II. Based on these previous experimental results, we rank the transcription rates in the following order: WT Pol II (C template) > R512A mutant (C template) > WT Pol II (5caC template). Our MD simulations predict that R512A promotes translocation by restoring bridge helix kinking (Fig. 4*B*), suggesting the crossover step of 5caC over bridge helix by R512A mutant on 5caC is faster than that of WT Pol II. However, we note that R512 is not the only factor that contributes to the slowdown of transcription in the 5caC system. Other factors such as the hydrogen bonding between 5caC and Q531 will also restrain the base at the midway position to across the bridge helix; thus, the R512A mutation alone will be insufficient to fully restore the transcription rate to the level of unmodified C. Therefore, the rate of R512A mutant (5caC template) is likely in between the rates of R512A mutant (C template) and WT Pol II (5caC template). Taken together from previous experimental data and our MD simulation data, we predict the transcription rates in following order: WT Pol II (C template) > R512A mutant (C template) > R512A mutant (5caC template) > WT Pol II (5caC template). We anticipate that these predictions can be tested in future experimental studies.

### The bases flanking 5caC have insignificant effect on transcription slowdown

In principle, the nucleotides flanking 5caC can impact transcription. In our MSM for 5caC, the template nucleotide directly upstream of 5caC (−1 site) is paired with the RNA nucleotide and does not exhibit specific interactions with the bridge helix or the hydrogen bond partners of 5caC. Therefore, dG in the −1 site is unlikely to impact transcription slowdown specific to 5caC. The +2 site nucleotide (dC) does not form hydrogen bonds with the 5caC base or any of the primary hydrogen bond partners of 5caC (Q531 or R512), indicating that the dC in the +2 site is unlikely to compete for hydrogen bonds with residues that restrict 5caC from fully reaching the +1 site. This might not be the case for other nucleobases. The possible impact of the flanking nucleotides on bridge helix kinking also deserves attention. We, therefore, conducted additional simulations to examine the impact of different −1 and +2 site nucleotides on the interactions of 5caC and bridge helix kinking (see the Experimental procedures section for details on the simulation setup and Fig. S9*A* for a complete list of sequences tested). In these simulations, the nucleotides flanking 5caC do not significantly alter bridge helix kinking (Fig. S9*B*) or the most significant hydrogen bonding partners of 5caC: R512 and Q531 (Fig. S9, *C* and *D*). Taken together, our simulations indicate that the effect of flanking nucleotides on bridge helix kinking or 5caC translocation might be less significant compared with the effect of 5caC itself. Therefore, the findings reported in our simulations are likely to be universal for different DNA sequences.

**Figure 5:**
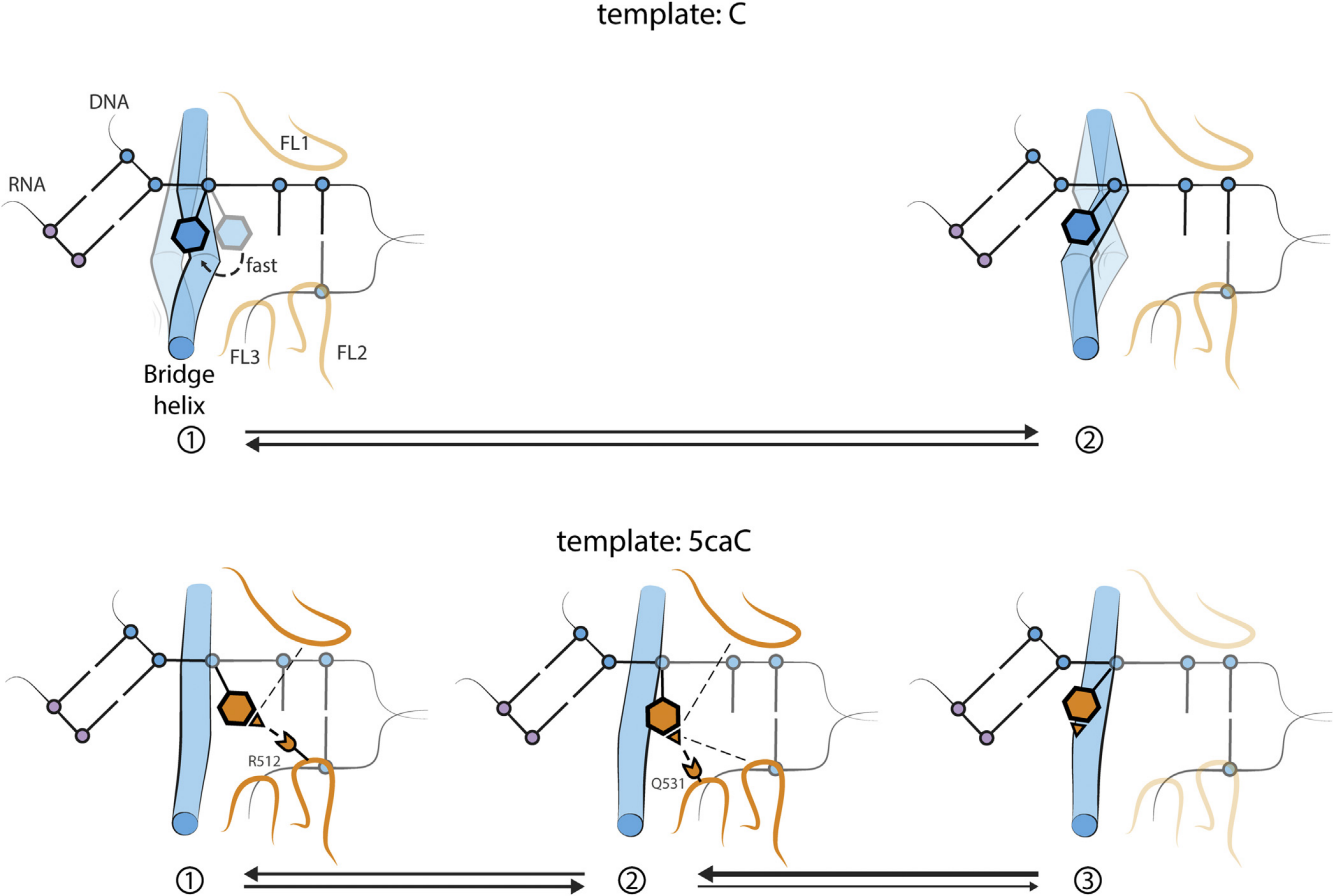
**A cartoon model summarizing the major results.***Top*, C rapidly transitions from the midway position and occupies the +1 template position, ready to pair with an incoming NTP. The bridge helix is kinking actively. *Bottom*, before reaching the +1 site, 5caC transitions through 2 metastable intermediates that exhibit hydrogen bonds with fork loops 1 to 3 (especially residues R512 in state 1 and Q531 in state 2) as well as hindered bridge helix kinking. 5caC, 5-carboxycytosine.

In this work, we analyzed MSMs based on MD simulations of the Pol II elongation complex with either 5caC or C as the template nucleotide. Figure 5 provides an overview of the major results of our study. The top row depicts an unmodified dC rapidly crossing the bridge helix into the +1 site, ready to pair with the incoming NTP. The bottom row illustrates 5caC restrained by multiple interactions with fork loops, most significantly, with residues R512 and Q531, as well as hindered bridge helix kinking. These interactions create 2 metastable states that make the +1 site less favorable for 5caC, slowing down elongation. We found that 5caC induces several effects that work together to reduce translocation. Two metastable states caused by 5caC prevent the template base from fully reaching the +1 site because of specific hydrogen bonds with residues R512 (Rpb2) and Q531 (Rpb2). Besides the two dominant hydrogen bonds, we observed that the carboxylic modification of 5caC transiently interacts with additional residues of Pol II, and these interactions account for as much hydrogen bonding as residues R512 and Q531 alone, further stabilizing the translocation intermediates. In addition, we observed bridge helix kinking taking place at four characteristic points, as well as a reduction in this kinking with 5caC, compared with C. We detected this effect over the whole length of the bridge helix, even at positions where 5caC does not contact it, including the transcription critical N-terminus hinge. This indicates that 5caC also reduces translocation indirectly. Alanine mutations of R512 and Q531 can restore the translocation in two ways: by changing the favorability of the metastable translocation intermediates and by reducing the barrier between the intermediates. In addition, the R512A mutation restores bridge helix kinking with 5caC, further promoting translocation.

Our work highlights the complexity of transcription, demonstrating how a single modification of a template DNA base, such a 5caC, can induce multiple effects that prevent translocation through both directly interacting with the protein residues and indirectly perturbing the conformational ensemble. Our work provides insights into how epigenetic modifications of genomic DNA can interfere with Pol II translocation and alter normal transcription.

## Experimental procedures

### System setup

The initial structure of the Pol II elongation complex with 5caC as the template nucleotide was built from the pretranslocated state from reference (11) and the crystal structure PDB ID: 4Y52 (9). The model contains a minimal Pol II elongation complex, in which the RNA strand is an 8-mer (3′-CCAGUGCG-5′), the DNA template strand is a 23-mer (3′-CGCACTGGXCTCGACTTTTAGCC-5′ where X is either C or 5caC), and the DNA nontemplate strand is a 10-mer (3′-GGCTAAAAGT-5′). The modified residue, 5caC, and its template nucleotides 16 to 22 were taken from PDB ID: 4Y52, which was aligned to nucleotides 12 to 18 in the previous model by heavy atoms regardless of 5caC. The residues of FL3 near Q531 (Rpb2 residues 528–535) were also taken from PDB ID: 4Y52 and aligned by the backbone heavy atoms.

### MD simulation setup

The MD simulations were conducted using Gromacs 5.0.4 (61) with Amber99SB force field (62) and OL15 correction (63). To produce the force field parameters for 5caC, the geometry was optimized, and the electrostatic potential charge was calculated at HF/6-31g∗ level of theory/basis set with Gaussian 09 package (64). The restricted electrostatic potential charge was fitted by the Antechamber package in AmberTools with a fixed charge value on the atoms O3′, H3T, O5′, and H5T (65). The missing bonded parameters were obtained from the general Amber force field (66).

The Pol II complexes were solvated in TIP4P water (67) in dodecahedral box vectors of length 17.6 nm and angles 60°, 90°, and 90°. To ensure that the system was electrostatically neutral, 70 and 71 molecules of water were replaced with sodium ions for the C and 5caC systems, respectively. The energy was minimized using the steepest descent algorithm for 10,000 steps, followed by a 1-ns constant number of particles (N), volume (V) and temperature (T) (NVT) MD simulation with a leapfrog integrator with a 2-fs timestep and restrains on heavy atom positions. The electrostatic interactions were cut off at 1.2 nm, and long-range electrostatic interactions were calculated using the Particle mesh Ewald method (68). van der Waals interactions were cut off at 1.1 nm. During the first 500 ps, the temperature of the system was brought to 298K with a V-rescale thermostat. Next, a 1-ns simulation with the pressure controlled by a Berendsen barostat was performed. The resulting conformations were used for production of NVT simulations (see the next section for more details on the production of MD simulations), and snapshots were saved at 100-ps intervals for subsequent MSM construction.

### MSM construction and validation

The conformations of the elongation complex sampled with MD simulations were used to construct MSMs of the template nucleotide transition over the bridge helix. The MSMs were constructed using the methods implemented in MSMBuilder 3.8.0 (69). Here, we followed our previously published protocol of MSM construction (52) (see below for details of each step): (a) Two rounds of MD sampling were performed to get a broad coverage of the conformational space of the post-translocation free energy basin. (b) The dimensionality of the sampled datasets was reduced using a tICA (53, 54, 55) of the 5caC dataset, based on a broad feature set involving parts of the system relevant to translocation. This feature set was optimized to be compatible with MSM construction. We built our MSM based on the tICA components of 5caC alone because the primary object of our study was the 5caC system and the C system served as a control simulation. Moreover, the 5caC system covered a broader range of conformations (Fig. 1, *C* and *D*), providing a better basis for state decomposition for the MSM construction. (c) The C and 5caC datasets were combined and projected onto the obtained tICA coordinates and clustered with a K-centers algorithm (57). The tICA correlation time and the number of clusters were optimized with a cross-validation scheme based on the GMRQ (56). In the cross-validation scheme, we repeatedly split our total data into two sets: training and testing. The training set is used to estimate the MSM with the given parameters, the GMRQ is then calculated using the testing set. A high GMRQ score signifies that the trained model can faithfully predict the slowest timescales, without overfitting the data and allows us to robustly select parameters, such as tICA lag time or the number of microstates. (d) The MSMs for C and 5caC were estimated separately by projecting the individual datasets onto the clusters. The resulting microstate-MSMs were validated with a residence probability test. (e) The MSMs were coarse-grained using spectral clustering to interpret the biological mechanism.

#### MD sampling

Two rounds of MD simulations were performed: initially, five replicas of each starting structure (with the template base in the +1 site and midway) were allowed to evolve for 100 ns with different initial velocities. After discarding the first 20 ns of each trajectory, the remaining conformations were clustered using the K-centers algorithm (57) into 50 clusters in the coordinates of the RMSD of atomic coordinates. The RMSD was measured using the C_α_ atoms of the bridge helix (residues 810–845 of Pol II subunit Rpb1), FL3 (residues 521–541 of Rpb2), heavy atoms of the two DNA bases flanking the template nucleotide, and the side chain of Q531 of Rpb2. The second round of simulation started with 50 structures chosen randomly from each cluster for each 5caC and C. In total, we performed fifty 100-ns MD simulations for each of the C and 5caC systems for the subsequent MSM construction. The MD trajectories of the 5caC system were used for subsequent tICA.

#### Feature selection as input to the tICA

The MSMs were constructed in the reduced space obtained through the tICA using interatomic distances as input features. The initial feature set was defined as a broad set of atom pairs from structural elements in the vicinity of the active site (7211 atom pairs), which were previously reported to play an important role in RNA polymerase translocation (11, 12): the bridge helix (Rpb1 812–844), the trigger loop (Rpb1 1049–1107), FL3 (Rpb2 528–532), the residues of Rpb2 within 1.2 nm of the 5caC in the initial structure, the 3′-RNA nucleotide, and the two template DNA nucleotides flanking 5caC. However, this initial set of input features to the tICA vectors resulted in a disconnected projection even onto the first two time-lagged independent components. Thus, this initial set of input features was further contracted by iteratively removing atom pairs until a visually connected tICA projection was achieved. As a result, an updated set of input features containing 4107 atom pairs was obtained. Next, the input features were further optimized by removing distances with negligible contributions to the variation of the tICA space. This was achieved by selecting 1000, 600, and 400 atom-pair distances with the largest contribution (*i.e.*, highest magnitude) to the tICA eigenvector components of tIC1, tIC2, and tIC3, respectively. Finally, these three sets of atom-pair distances were merged into a unique set of features that consisted of 1481 distances between pairs of atoms (Fig. S2 and Supplemental Data S1 for a complete list of atom pairs).

#### Choice of hyperparameters

The tICA correlation time and the number of clusters were selected using a cross-validation scheme based on the GMRQ (56). For each of the parameter values, the set of all MD trajectories were split five times with a 1:1 ratio to generate training and test sets. Figure S3*A* shows the distribution of the GMRQ overlaps between various choices of tICA correlation time; based on this, the correlation time value was selected as 2 ns because it can produce the highest GMRQ.

#### Microstate-MSM construction and validation

To better compare the translocation dynamics of the 5caC and C systems, a common set of 800 microstates was produced with K-centers clustering (57) on the merged datasets using the tICA parameters identified in the previous section. Again, the optimal number of clusters was chosen based on the highest GMRQ score (Fig. S3*B*). Next, the conformations of 5caC were assigned to this set of 800 clusters, resulting in a 388-microstate-MSM. The MSM lag time cannot be selected based on GMRQ; therefore, after estimating the top timescales at various lag times, we select the lag time at which the timescales are relatively constant while favoring smaller lag time. Therefore, the lag time was chosen to be 45 ns, as illustrated in Figure S3*C*. The microstate-MSM was validated using a residence probability test (Fig. S4*A*). The residence probability test compares the state population after a given number of MSM steps (orange curves in Fig. S4) to the simulation data (blue in Fig. S4). If the model quality is good, the two curves are expected to lie within the confidence interval. The 12 most populated microstates were selected and propagated twice using the transition probability matrix. The MD-based residence probability was calculated using the given lag time (0, 45, and 90 ns) for each of the 12 microstates with at least 4.5 ns between the initial frames and using a smoothing window of 1 ns. The residence probability test shows that the MSM can approximate the sampled dynamics of 5caC reasonably well. To facilitate the comparison between 5caC and C, the MD conformations of C were also assigned to the set of 800 clusters, which produced a 373 microstate-MSM (lag time at 45 ns, Fig. S3*D*). Among the two microstate-MSMs, 140 microstates were populated by both the 5caC and C systems. The MSM for C was also validated with a residence probability test (Fig. S4*B*).

#### Kinetic lumping to macrostate MSMs

To facilitate the interpretation of the biological mechanism, the microstate-MSM for C and 5caC was coarse-grained based on spectral clustering (as implemented in SciPy 1.5.0 (70)). The number of coarse-grained states was chosen based on the gap between the top eigenvalues of the transition probability matrix: three states for 5caC and two states for C.

### Estimation of transition pathways and mean first-passage times

The microstate-MSMs were used to estimate the transition pathways and mean first-passage times between the macrostates. Equilibrium populations and SDs of macrostates and the mean first-passage times were estimated from ten Markov chain Monte Carlo (MCMC) trajectories of 10^5^ steps generated from the corresponding transition probability matrix, discarding the first 20% of steps. The transition pathways for 5caC (Fig. 1, *C* and *D*) were calculated based on 95% of the reactive flux between macrostates as implemented in PyEMMA 2.5.7 (71).

### Estimation of hydrogen bond probabilities

The hydrogen bonds were calculated using Gromacs 5.0.4 (61). Gromacs uses a geometric criterion to determine if a hydrogen bond is present. The hydrogen bond is present in a structure if the distance between the donor and the acceptor atom is less than 0.35 nm and the hydrogen atom in the bond deviates less than 30° from the line connecting the donor and acceptor. The mean and SD of the hydrogen bond population in macrostates were estimated from ten MCMC trajectories of 10^5^ steps sampled from the corresponding microstate-MSM. Side chains of some amino acids, such as arginine, can form multiple hydrogen bonds simultaneously, and such interactions contributed proportionally to the reported hydrogen-bond population between residues.

### Evaluation of bridge helix kinking

Kinking of the bridge helix was calculated with Kink Finder (58). The bridge helix was classified as kinked if it had at least one kink with a wobble angle greater than 35°. This value was chosen based on the minimum between two evident peaks in kink angle distribution of residues 825 to 828 (Fig. S5). The bridge helix can exhibit multiple kinks simultaneously; however, more than one kink does not increase the reported population of a kinked bridge helix. The mean and 95% of the bootstrap distribution of the kinking percentage was calculated using the same MCMC trajectories that were used for the calculation of the hydrogen bonds. The odds ratio was calculated from the same MCMC trajectories using the formula OR = (B_s_/B_k_)/(NB_s_/NB_k_), where B_s_ represents the population of the given macrostate with a straight bridge helix and a specific hydrogen bond and B_k_ represents the population of the given macrostate with a kinked bridge helix and the specific hydrogen bond; similarly, NB_s_ and NB_k_ represent populations with a straight and kinked bridge helix, respectively, that do not exhibit the hydrogen bond.

### Pol II mutant MD simulations

To evaluate the effect of R512A and Q531A mutations, additional MD simulations were initiated from structures close to the barrier between the macrostates. By selecting a set of structures with approximately equal probability to transition to either side of a barrier (given by the MSM), the WT simulations would exhibit a symmetric distribution along the reaction coordinate relative to the starting structures; therefore, the effect of the mutation can be asserted from the change in the mutant distribution relative to the WT. For the R512A mutant, ten structures were chosen from the 5caC MSM to initiate the mutant MD simulations: Five microstates with the highest probability to transition into macrostate 2 were selected from macrostate 1, and a single structure was randomly chosen from each of these microstates. Five more structures with the highest probability to transition into macrostate 1 were selected from macrostate 2. Next, the R512 residue of Rpb2 was mutated to alanine. The MD simulations were set up similarly to the initial simulation set up and were computed up to 100 ns after the equilibration phase. In total, ten WT simulations and ten R512A simulations were conducted. Similarly, ten structures were selected from the 5caC MSM to represent the transition region between macrostates 2 and 3, and subsequently, residue Q531 was mutated to alanine. Again, ten WT systems for 100 ns and ten Q531A systems were simulated. The percentage of straight bridge helix conformations in the mutant set of simulations was reported as the mean of the ten trajectories and 95% of the bootstrap distribution from resampling blocks of 4 ns 1000 times with replacement.

### Simulation setup of the DNA mutant variants

Because of the two midway states, state 1 displays the highest population of straight bridge helix conformations (Fig. 2*B*), two representative structures were selected from state 1 of 5caC, containing a straight bridge helix and a kinked one. We then introduced all possible single mutations of the bases in the −1 and +2 sites (see Fig. S9*A* for a complete list of sequences) and conducted additional 50-ns simulations. These simulations were repeated three times with different initial velocities for each starting structure to evaluate the impact of these mutations—a total of six simulations for each mutant. To control for the effect of the mutations, we ran additional simulations starting from the same structures (repeated three times) without changing the DNA sequence. To measure the effect of the flanking bases on the hydrogen bond between 5caC and Q531, we ran another round of simulations starting with two structures selected from macrostate 2: one with the hydrogen bond and the other lacking the hydrogen bond. Again, each structure was simulated for 50 ns with 3 repeats. For control, six more simulations were carried out without changes to the DNA sequence. A total of 84 ∗ 50-ns trajectories were conducted to address the effect of the template DNA nucleotides flanking 5caC.

## Data availability

Data are available from the corresponding author upon request.

## Supporting information

This article contains supporting information.

## Conflict of interest

The authors declare that they have no conflicts of interest with the contents of this article.
