# Supplementary material for: A comprehensive mechanism for 5-carboxylcytosine-induced transcriptional pausing revealed by Markov state models
Source: J Biol Chem. 2021 May 13;296:100735. doi: 10.1016/j.jbc.2021.100735 (PMC8191312; doi:10.1016/j.jbc.2021.100735)
Supplement: Figures S1 to S9 and Tables S1 to S4 [file mmc1.pdf]

Supporting Information for:

A comprehensive mechanism for 5-carboxylcytosine induced transcriptional pausing revealed by Markov state models

Kirill A. Konovalov[1,2], Wei Wang[1], Guo Wang[1], Eshani C. Goonetilleke[1,2], Xin Gao[3], Dong Wang[4], Xuhui Huang*[1,2]

[1]Department of Chemistry, State Key Laboratory of Molecular Neuroscience, The Hong Kong University of Science and Technology, Kowloon, Hong Kong
[2]Hong Kong Center for Neurodegenerative Diseases, Hong Kong Science Park, Hong Kong
[3]Computational Bioscience Research Center, Computer, Electrical and Mathematical Sciences and Engineering Division, King Abdullah University of Science and Technology (KAUST), Thuwal 23955-6900, Saudi Arabia.
[4]Division of Pharmaceutical Sciences, Skaggs School of Pharmacy and Pharmaceutical Sciences; Department of Cellular and Molecular Medicine, School of Medicine; Department of Chemistry and Biochemistry, University of California, San Diego, La Jolla, California 92093, United States

* To whom correspondence should be addressed. Email: xuhuihuang@ust.hk

Supplementary Figures:

A

Bridge Helix:

```
T.thermophilus 1068 - LEYFISSHGARKGGADTALRTADSGYLTRKLVDVT - 1102
M.jannaschii   1262 - TEFFFHAMGGREGLVDQAVRTAQSGYMQRRLINAL - 1297
S.cerevisiae    811 - QEFFFHAMGGREGLIDTAVKTAETGYIQRRLVKAL - 845
                       *:*: : *.*:*  * *:.**::**: *.*:..
```

B

Fork Loop 2:

```
T.thermophilus  415 - GGLTRERAGFDVRDVHRTHYGRICPVETPEGANIG - 450
M.jannaschii    442 - SPLSRSQPHFEARELHGTHWGKICPSETPEGPNCG - 477
S.cerevisiae    500 - TPIGRDGKLAKPRQLHNTHWGLVCPAETPEGQACG - 534
                        : *.    . *::* **:* :** *****   *
```

Fig S1. Sequence conservation of bacterial, archaeal and eukaryotic bridge helix and fork loop 2. A. Alignment of sequences corresponding to the bridge helix. **B.** Alignment of fork loop 2 sequences, the position corresponding to residue 512 of the yeast Rpb2 is highlighted by a grey box; The alignment codes are: "*" stands for a perfect match, ":" – strong similarity, "." – weak similarity.

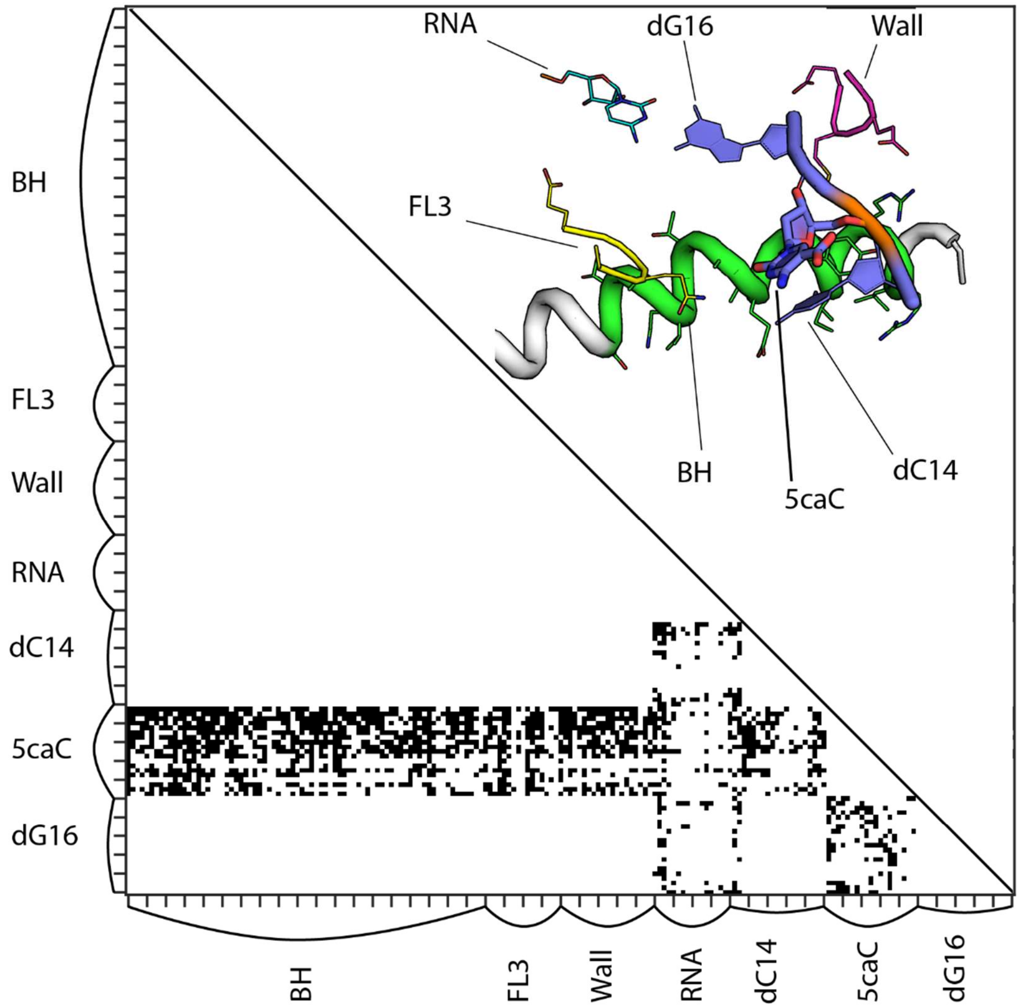

Fig S2. Input feature set for tICA. The interatomic distances between heavy atoms were computed between structural elements of Pol II as shown. Bridge helix: residues 826-840 (Rpb1); FL3: 829-832 (Rpb2), Wall: 1131-1133 (Rpb2); RNA – 3’ terminal RNA nucleotide (C); dC14 – the template DNA nucleotide directly downstream of 5caC; 5caC – the template nucleotide; dG16 – the template DNA nucleotide directly upstream of 5caC. See supplementary data 1 for the complete list of atom pairs.

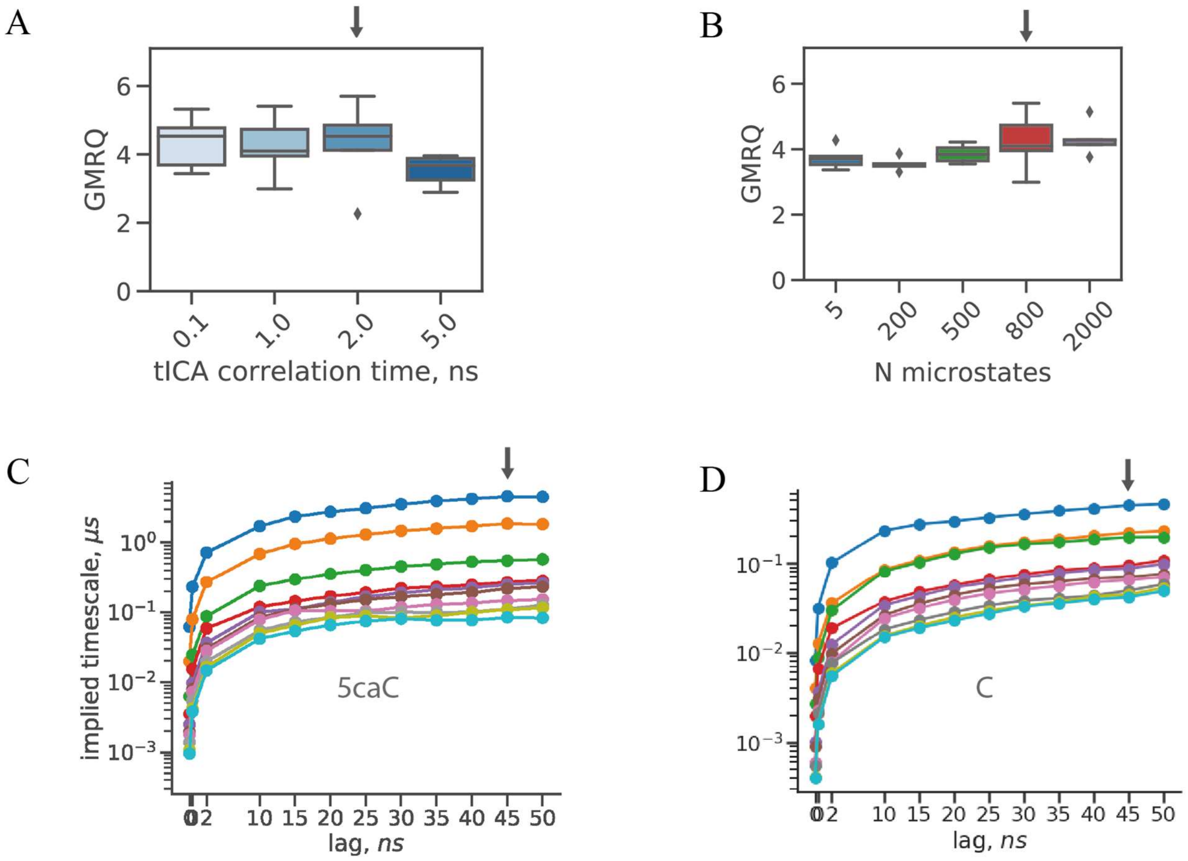

Figure S3. Markov State Model Parameter Choice. A. GMRQ based tICA correlation time selection for the 5caC model. Each box depicts distributions of GMRQ obtained from randomly splitting the data five times. The parameter choice producing the highest value is highlighted with an arrow. **B.** GMRQ based selection of the number of clusters for the combined dataset **C.** Convergence test based on the implied timescale of the TPM for 5caC simulations. **D.** Convergence test based on the implied timescale of the TPM for C. The black arrows point to the final parameter choice.

A

B

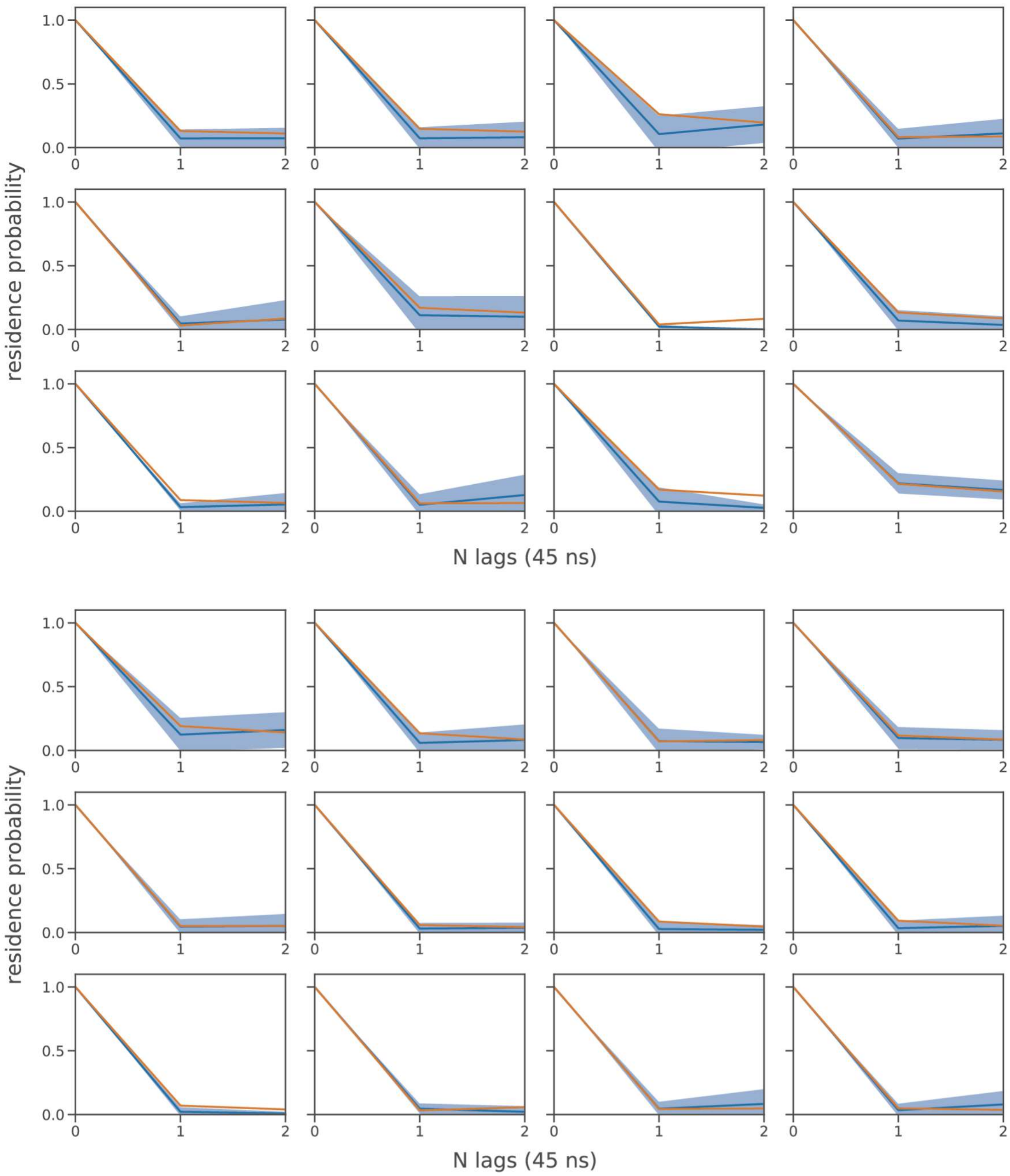

Fig S4. Validation of the MSMs. A. Residence probability test of the top 12 populated microstates of the MSM for 5caC. **B.** Residence probability test of the top 12 populated microstates of the MSM for C. The MSM based probability is shown in orange. The MD based probability is shown in blue. The standard deviation interval (shown in transparent blue) was calculated from the distribution generated by resampling the initial set of trajectories 500 times with replacement.

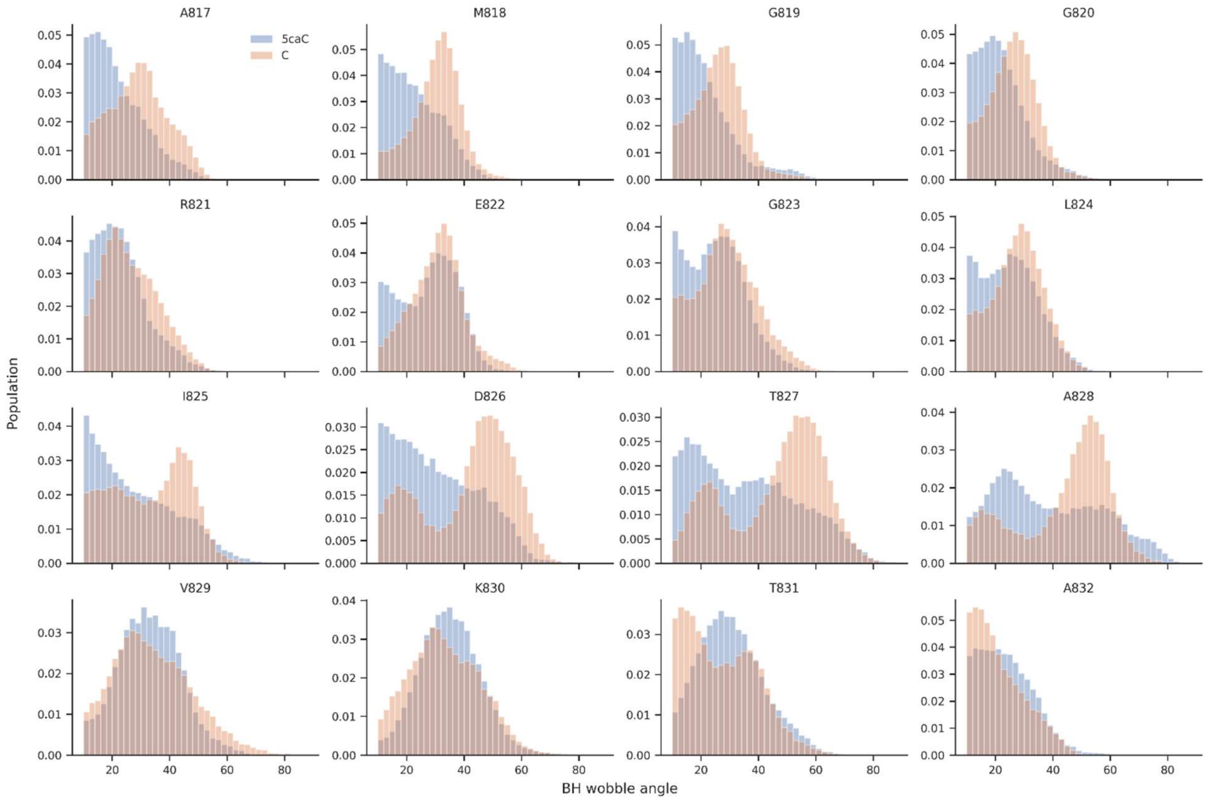

Figure S5. Normalized kinking angle histograms of individual bridge helix residues. These distributions are based on wobble angles measured at given positions without imposing the kinked/straight cutoff.

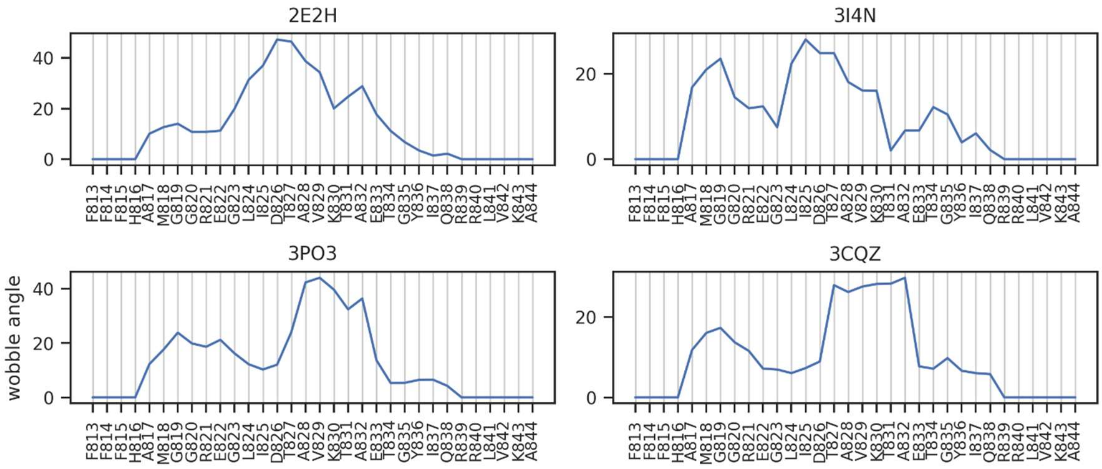

Fig S6. Bridge Helix kinking in crystal structures of Pol II. The panels correspond to the crystal structure with the PDB id in the title. The wobble angle of the structure measured along the bridge helix is shown.

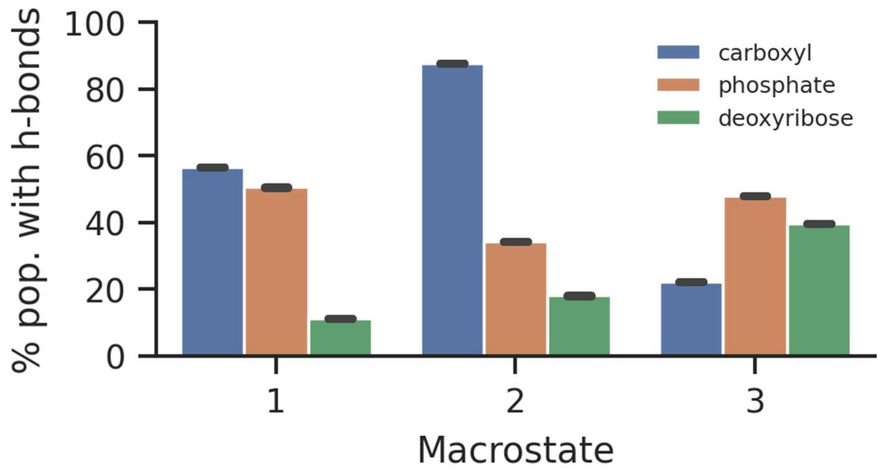

Figure S7. Total hydrogen bonding in macrostates of 5caC. The height of the bars reflects the percentage of the population in the macrostate that displays a hydrogen bond with the given group. Hydrogen bonding with deoxyribose involves all oxygen atoms of the moiety, similarly, all oxygen atoms were included for the phosphate and carboxyl groups. Error bars represent 95% confidence intervals of the bootstrap distribution.

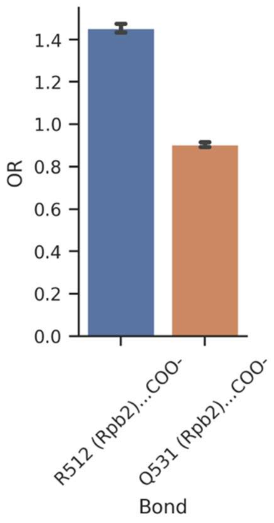

Fig S8. Odds Ratio of hydrogen bonds occurring with straight bridge helix, relative to kinked bridge helix in the 5caC model. The height of the bars reflects the odds of finding a hydrogen bond with the bridge helix in straight conformation versus kinked. The first bar shows the odds of finding the hydrogen bond between R512 and 5caC in macrostate 1, the second bar is calculated for the hydrogen bond between Q531 and 5caC in macrostate 2. Error bars represent 95% confidence intervals of the bootstrap distribution.

A

Label		Sequence: +2:+1:-1
1	dC	dC:dC:dG
2	5caC	dC:5caC:dG
3	5caC	**dG**:5caC:dG
4	5caC	**dA**:5caC:dG
5	5caC	**dT**:5caC:dG
6	5caC	dC:5caC:**dC**
7	5caC	dC:5caC:**dA**
8	5caC	dC:5caC:**dT**

B

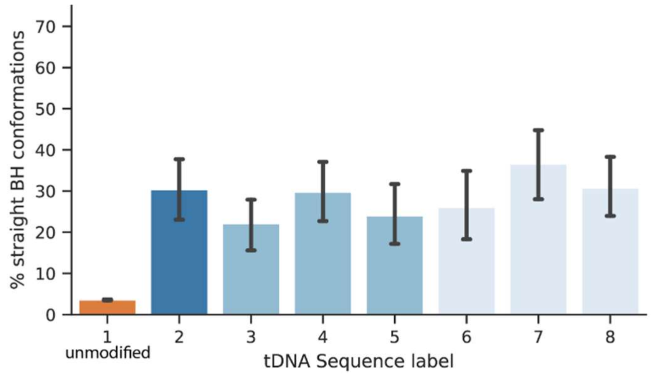

C

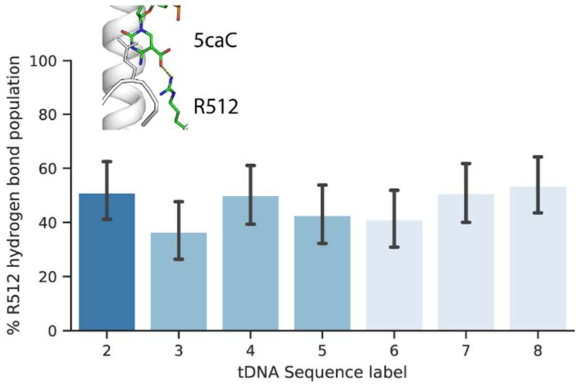

D

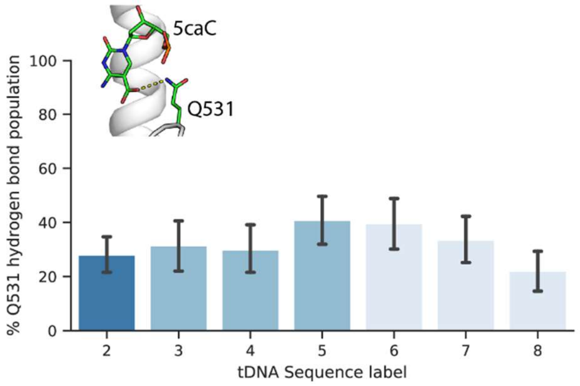

Figure S9. Effect of template nucleotides flanking the +1 site on transcription. A. Sequences of the template DNA (sites +2, +1, and -1) used to test the effect of the nucleotides flanking 5caC. The mutations to the original sequence are highlighted in bold. **B.** Bridge helix kinking in MD simulations starting from macrostate 1. The bar corresponding to sequence 1 (unmodified C) was reproduced from figure 2B. **C.** Percentage of structures with 5caC···R512 hydrogen bond in simulations starting from macrostate 1. (same as panel B) **D.** Percentage of structures with 5caC···Q531 hydrogen bond in simulations starting from macrostate 2. The error bars represent 95% of the bootstrap distribution, samples were generated from blocks of 4 ns. The lighter shade of blue in the bar plots is used to highlight the -1 site mutations, while the darker shade highlights the +2 site mutations.

Table S1. Equilibrium populations of the macrostates. The mean and the error (one standard deviation) are calculated from 10 MC trajectories of length 4.5ms.

State	Equilibrium population (%)
C	
1	62.4 ± 0.2
2	37.6 ± 0.2
5caC	
1	37.5 ± 0.2
2	48.4 ± 0.2
3	14.1 ± 0.1

Table S2. Transition timescales between macrostates. The timescales are reported in units of microseconds. The mean and the error (one standard deviation) are calculated from 10 Monte-Carlo trajectories of length 4.5ms.

C

State (*sink)*		1	2
(source)	1	-	0.8 ± 0.00
	2	0.5 ± 0.00	-

5caC

State (*sink)*		1	2	3
(source)	*1*	-	3.1 ± 0.06	22.0 ± 0.54
	2	4.2 ± 0.07	-	16.8 ± 0.39
	3	7.7 ± 0.07	3.0 ± 0.04	-

Table S3. Hydrogen bonding of the template nucleotide in the C system in macrostates. The hydrogen bonding partner is given in the first column (assumed to be the sidechain atoms, unless stated otherwise), the part of the template nucleotide is given in the second column (with deoxyribose being atoms O3’ and O5’combined and phosphate including both of the oxygen atoms). The percentage of the equilibrium population of the macrostate is given in the last column. The mean and the error estimate (given as the standard deviation) are calculated from sampling 10 Monte-Carlo trajectories of length 4.5 ms. The values depicted in Fig. 3C of the main text are highlighted in bold.

Pol II residue	template nucleotide group	Population (%)
	Macrostate 1	
T831	N4	7.5 ± 0.17
K332	phosphate	10.1 ± 0.21
S474 (Rpb2)	phosphate	31.0 ± 0.34
**Q531 (Rpb2)**	**phosphate**	**28.0 ± 0.38**
	Macrostate 2	
S474 (Rpb2)	phosphate	8.4 ± 0.24
**Q531 (Rpb2)**	**phosphate**	**13.3 ± 0.25**
R839	deoxyribose	32.7 ± 0.84

Table S4. Hydrogen bonding of the template nucleotide in the 5caC system in macrostates. The hydrogen bonding partner of the template nucleotide is given in the first column (assumed to be the sidechain, unless stated otherwise), the part of the template nucleotide is given in the second column (with COO^- being the carboxylic group of the 5caC, deoxyribose being atoms O3' and O5'combined and phosphate including hydrogen bonds with both of the oxygen atoms). The percentage of the equilibrium population of the macrostate is given in the last column. The mean and the error estimate (given as the standard deviation) are calculated from sampling 10 Monte-Carlo trajectories of length 4.5 ms. The values depicted in Fig. 3D of the main text are highlighted in bold.

Pol II residue	template nucleotide group	Population (%)
	Macrostate 1	
K332	phosphate	13.7 ± 0.9
R337	phosphate	11.1 ± 0.68
S474 (Rpb2)	COO-	8.2 ± 0.51
R504 (Rpb2)	COO-	5.9 ± 0.39
K507 (Rpb2)	phosphate	6.0 ± 0.45
**R512 (Rpb2)**	**COO-**	**20.7 ± 1.28**
Q531 (Rpb2)	COO-	7.8 ± 0.45
A828 (backbone)	N4	13.8 ± 0.94
T831	N3	13.3 ± 0.94
T831	O2	11.5 ± 0.73
R839	phosphate	6.1 ± 0.0
R840	phosphate	8.8 ± 0.52
	Macrostate 2	
R476 (Rpb2)	COO-	7.6 ± 0.26
K507 (Rpb2)	phosphate	6.6 ± 0.33
K510 (Rpb2)	COO-	8.0 ± 0.28
**Q531 (Rpb2)**	**COO-**	**44.4 ± 1.07**
Q531 (Rpb2)	phosphate	9.4 ± 0.45
Q531 (Rpb2) (backbone)	COO-	9.1 ± 0.27

T831	COO-	15.0 ± 0.73
T831	N3	13.2 ± 0.43
T831	N4	8.8 ± 0.2
R839	phosphate	12.9 ± 0.0
R839	deoxyribose	16.6 ± 0.0
Macrostate 3		
**Q531 (Rpb2)**	**COO-**	**5.8 ± 0.72**
Q531 (Rpb2)	phosphate	53.8 ± 4.55
Q531 (Rpb2) (backbone)	COO-	13.0 ± 0.99
R839	O2	12.8 ± 0.85
R839	deoxyribose	40.9 ± 0.0
